# First person – Phuong Le and Jeanne Quinn

**DOI:** 10.1242/bio.061871

**Published:** 2025-01-08

## Abstract

First Person is a series of interviews with the first authors of a selection of papers published in Biology Open, helping researchers promote themselves alongside their papers. Phuong Le and Jeanne Quinn are co-first authors on ‘ α-catenin phosphorylation is elevated during mitosis to resist apical rounding and epithelial barrier leak’, published in BiO. Phuong is a research technologist in the lab of Dr Cara J. Gottardi at ​the Departments of Pulmonary Medicine and Cell and Developmental Biology, Northwestern University Feinberg School of Medicine, Chicago, IL, USA, and Jeanne is a MD-PhD student in the same lab. They are investigating how α-catenin phosphorylation restrains mitotic cell rounding in the apical direction, strengthening interactions between dividing and non-dividing neighbors to limit epithelial barrier leak.


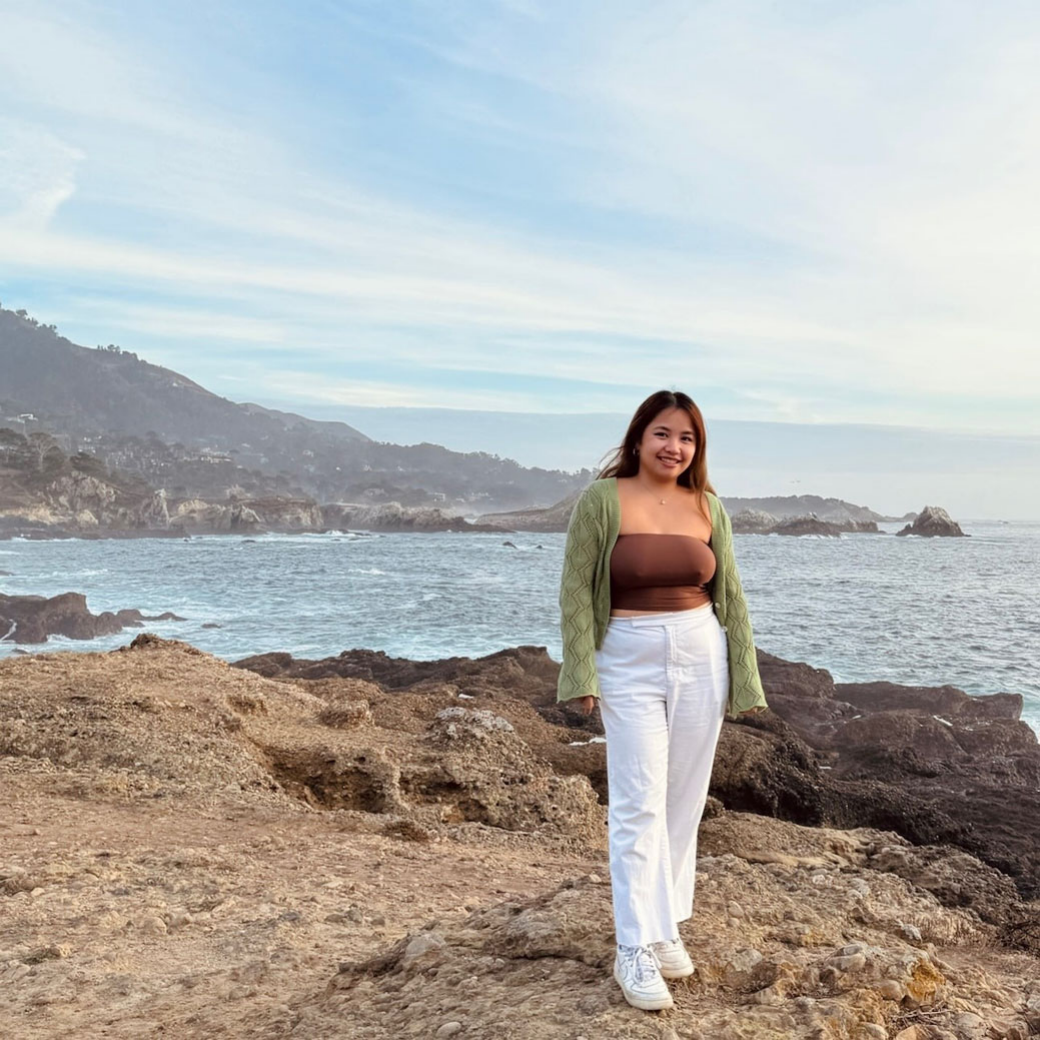


**Phuong Le**

**Describe your scientific journey and your current research focus**

P.L.: During my undergraduate, I was interested in the biophysiology of Parkinson's disease, my grandfather's diagnosis, and worked on validating metabolic probes to characterize the condition. The reward of refining hand dexterity during murine surgery, troubleshooting experimental design and collaborating with scientific professionals directed my efforts and goals towards furthering medical research. As a result, I graduated with a degree in the biological sciences with a concentration in cell and developmental biology. Since my post-baccalaureate, I have been working under Dr Cara J. Gottardi at Northwestern University to understand the role of cell adhesion and signaling in alveolar stem cell regeneration and differentiation.

J.Q.: My scientific journey has spanned several disciplines and approaches, providing essential tools and perspectives that shaped my approach to studying disease progression. Early in my career, I learned rigorous screening methodologies through phage-display projects and explored biophysical techniques in protein aggregation studies. Over time, I became increasingly drawn to imaging as a tool to investigate how molecular mechanisms drive cellular behaviors. My current research combines fundamental cell biology with unbiased image analysis to examine how cell adhesion is dynamically regulated in homeostasis and dysregulated in disease progression.

**Who or what inspired you to become a scientist?**

P.L.: Fascinated by the ingenuity of medicine, my budding curiosity in medical research began in a sixth-grade class trip to witness a live sternotomy. While most classmates looked away, I stared wide-eyed at the screen at the fact that the brutal bone sawing could increase human longevity. My skepticism directed my eagerness to immerse myself in the process of scientific pioneering to understand human physiology.

J.Q.: I grew up surrounded by research and have been interested in science from an early age. Mentors such as Dr George Smith inspired me to pursue research, demonstrating how scientific curiosity and a commitment to the fair and open sharing of technology can drive meaningful progress in science.

**How would you explain the main finding of your paper?**

Dysregulated cell adhesion and division are features of many disease states, but how cells dynamically maintain adhesion is not well understood. We found that the adhesion protein α-catenin has a key modification that allows dividing cells to stay better connected to their neighbors, helping the tissue stick together during mechanical stress.

… the adhesion protein α-catenin has a key modification that allows dividing cells to stay better connected to their neighbors, helping the tissue stick together during mechanical stress

**What are the potential implications of this finding for your field of research?**

Our findings support the idea that cells dynamically regulate adhesion during mitosis. Understanding how these adhesive programs function across dividing and neighboring cells could reveal molecular targets or therapeutic strategies for diseases where barrier function is compromised, such as fibrosis or cancer.


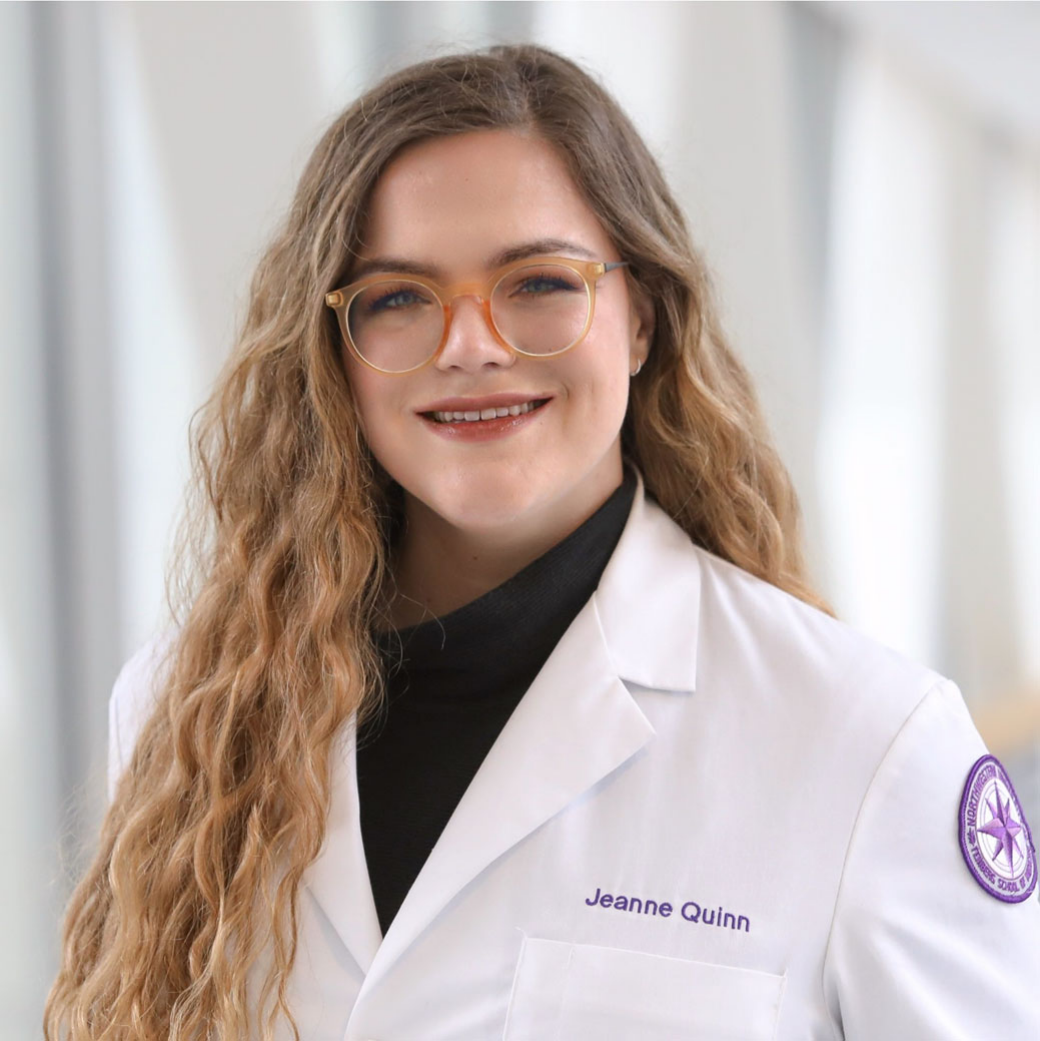


**Jeanne Quinn**

**Figure:**
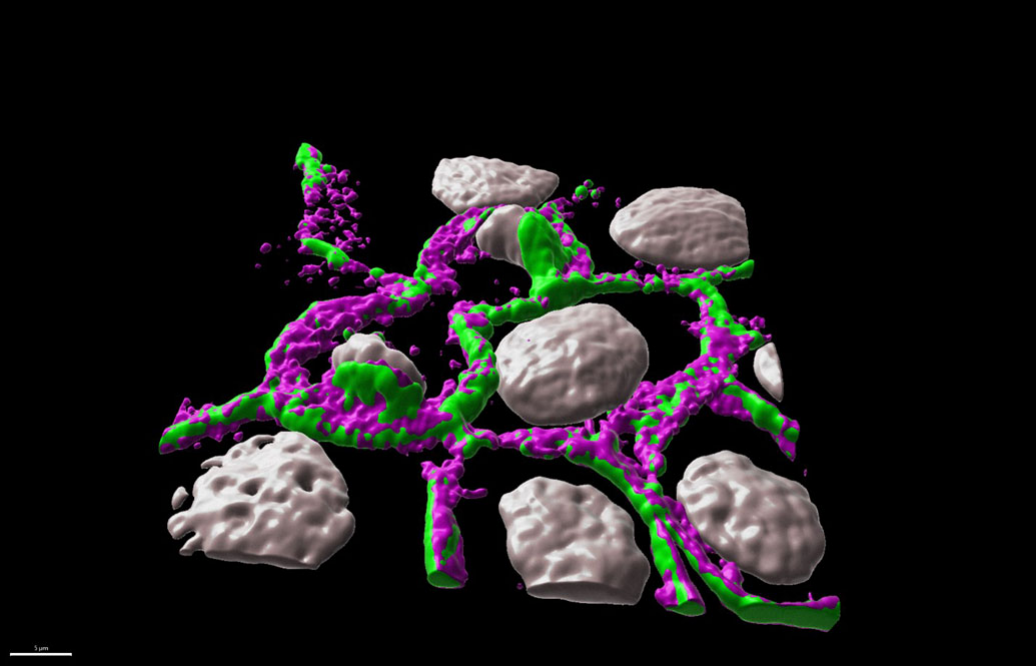
**The phosphorylated form of α-catenin localizes to the apical portion of adherens junctions.** The image shows 3D surface rendering using Imaris software of a confocal image of a dividing kidney epithelial cell line (Madin–Darby canine kidney or MDCK cells). Phosphorylated α-catenin is shown in magenta and adherens junctions are in green.

**Which part of this research project was the most rewarding?**

P.L.: The most rewarding part of this project was presenting meaningful data from seemingly mild visual differences. It was fulfilling to collaborate with physicists, cell biologists and microscopy experts to troubleshoot our challenges and imagine cell dynamics in different contexts. It was encouraging to discuss with other scientists interested in our insights. Soon, our findings will be applied and presented in a mouse model.

J.Q.: I found the range of techniques used to be the most rewarding aspect of this work. I collaborated with researchers in my lab and across campus to come up with plans for how to round out observations, using specialized image analysis workflows to quantitatively express biological phenomena that we can usually only appreciate as qualitative pictures.

**What do you enjoy most about being an early-career researcher?**

P.L.: As an early-career researcher, I find it fruitful to engage and collaborate with professionals across disciplines (clinicians, biologists, physicists and bioinformaticians) to investigate and interpret results. As a team, we coordinated strengths and weaknesses to promote intellectual creativity and well-rounded models.

J.Q.: I appreciate being able to pursue a central scientific question, while also having the flexibility to investigate offshoots as they arise. I get to balance directed, hypothesis-driven work with opportunities to develop new skills or learn from researchers in different fields. This stage of my career provides a unique combination of focused discovery and broad exploration.

**What piece of advice would you give to the next generation of researchers?**

P.L.: Maintaining a resilience and growth mindset is key to success. Setbacks are common in the field of research, but it is important to maintain optimism, proactiveness and visualization of the end goal. It is also important to identify and build a strong support system throughout your journey.

J.Q.: I have worked on projects where my interest in the topic or technique was my primary motivation, but I also recommend considering what skills you want to develop and identifying who can best support you in that growth. Find mentors who will help you navigate a career path that aligns with your strengths, interests and personalized goals.

Setbacks are common in the field of research, but it is important to maintain optimism, proactiveness and visualization of the end goal.

**What's next for you?**

P.L.: I am planning to matriculate to medical school with the intention to engage with academic medicine. I hope to continue to explore and apply stem cell signaling in the context of medical diseases.

J.Q.: I am completing my dissertation in the Gottardi lab, after which I will return to the clinic to finish my medical degree. My long-term goal is to combine clinical care and mechanistic research to better understand disease processes and improve patient outcomes.
